# Mycophenolic acid and 6-mercaptopurine both inhibit B-cell proliferation in granulomatosis with polyangiitis patients, whereas only mycophenolic acid inhibits B-cell IL-6 production

**DOI:** 10.1371/journal.pone.0235743

**Published:** 2020-07-09

**Authors:** Anouk von Borstel, Wayel H. Abdulahad, Gerjan Dekkema, Abraham Rutgers, Coen A. Stegeman, Johanna Veldman, Peter Heeringa, Jan Stephan Sanders

**Affiliations:** 1 Department of Internal Medicine, Division of Nephrology, University Medical Center Groningen, University of Groningen, Groningen, the Netherlands; 2 Department of Rheumatology and Clinical Immunology, University Medical Center Groningen, University of Groningen, Groningen, the Netherlands; 3 Department of Pathology and Medical Biology, University Medical Center Groningen, University of Groningen, Groningen, the Netherlands; University of South Florida St Petersburg, UNITED STATES

## Abstract

Granulomatosis with polyangiitis (GPA) is an autoimmune disease affecting mainly small blood vessels. B-cells are important in the GPA pathogenesis as precursors of autoantibody-producing cells but likely also contribute (auto)antibody-independently. This has been underlined by the effectiveness of B-cell-depletion (with Rituximab) in inducing and maintaining disease remission. Mycophenolate-mofetil (MMF) and azathioprine (AZA) are immunosuppressive therapies frequently used in GPA-patients. Interestingly, MMF-treated GPA-patients are more prone to relapses than AZA-treated patients, while little is known about the influence of these drugs on B-cells. We investigated whether MMF or AZA treatment (or their active compounds) alters the circulating B-cell subset distribution and has differential effects on *in vitro* B-cell proliferation and cytokine production in GPA-patients that might underlie the different relapse rate. Circulating B-cell subset frequencies were determined in samples from AZA-treated (n = 13), MMF-treated (n = 12), untreated GPA-patients (n = 19) and matched HCs (n = 41). To determine the *ex vivo* effects of the active compounds of MMF and AZA, MPA and 6-MP respectively, on B-cell proliferation and cytokine production, PBMCs of untreated GPA-patients (n = 29) and matched HCs (n = 30) were cultured for 3-days in the presence of CpG-oligodeoxynucleotides (CpG) with MPA or 6-MP. After restimulation (with phorbol myristate acetate, calcium-ionophore), cytokine-positive B-cell frequencies were measured. Finally, to assess the effect of MMF or AZA treatment on *in vitro* B-cell proliferation and cytokine production, PBMCs of MMF-treated (n = 18), and AZA-treated patients (n = 28) and HCs (n = 41) were cultured with CpG. The memory B-cell frequency was increased in AZA- compared to MMF-treated patients, while no other subset was different. The active compounds of MMF and AZA showed *in vitro* that MPA decreased B-cell proliferation in GPA-patients and HCs. B-cell proliferation in MMF- and AZA-treated patients was not different. Finally, the IL-6^+^ B-cell frequency was decreased by MPA compared to 6-MP. No differences in IL-10^+^, IL-6^+^ or TNFα^+^ B-cell proportions or proliferation were found in MMF- and AZA-treated patients. Our results indicate that MMF could be superior to AZA in inhibiting B-cell cytokine production in GPA-patients. Future studies should assess the effects of these immunosuppressive drugs on other immune cells to elucidate mechanisms underlying the potential differences in relapse rates.

## Introduction

Granulomatosis with polyangiitis (GPA) is a systemic autoimmune disease characterized by inflammation of small- to medium-sized blood vessels. GPA is associated with the presence of anti-neutrophil cytoplasmic antibodies (ANCA) mainly directed against proteinase 3 [1]. Patients with GPA frequently suffer from severe disease relapses that increase the disease burden.

Patients suffering from autoimmune diseases such as GPA and systemic lupus erythematosus (SLE) receive induction- and maintenance immunosuppressive therapy to treat active disease and prevent disease relapses, respectively. Remission maintenance therapy often consists of mycophenolate mofetil (MMF) or azathioprine (AZA) combined with glucocorticoids. The active compounds of both MMF and AZA inhibit purine nucleotide synthesis, which is important for DNA synthesis and lymphocyte proliferation [2]. The active compound of MMF, mycophenolic acid (MPA), inhibits the enzyme inosine monophosphate dehydrogenase 2 (IMPDH2), an isotype which is specifically upregulated in activated lymphocytes. The active compound of AZA, 6-mercaptopurine (6-MP), non-selectively inhibits IMPDH resulting in inhibition of all activated immune cells [3–5].

B cells play an important role in the GPA pathogenesis as precursors of ANCA-producing plasma cells. Importantly, B cells also exert antibody (Ab)-independent properties such as antigen presentation [6] and cytokine production [7]. These Ab-independent B cell functions gained more interest in GPA since rituximab, a CD20^+^ B cell depleting monoclonal Ab, was proven effective in inducing and maintaining disease remission [8,9]. Although the ANCA-producing CD20^-^ plasma cells are not targeted by rituximab, a gradual decrease in serum ANCA is seen upon B cell depletion by rituximab and induction of remission in GPA patients [10]. This finding indicates that the sole presence of ANCA in the circulation, in the absence of CD20^+^ B cells, does not induce GPA reactivation and seems to underline the importance of Ab-independent functions of B cells in the GPA pathogenesis [9]. Indeed, we previously demonstrated several alterations in the B cell compartment of GPA patients [11–13]. An altered B cell subset distribution in GPA patients was found, characterized by increased circulating naïve and decreased memory B cell and regulatory B cell (Breg) frequencies [11]. Moreover, Bregs of GPA patients correlated inversely with T helper (Th) 17 cells and showed decreased suppression of interleukin (IL)-17-producing Th cells compared to HCs [12]. Lastly, we recently reported that B cells of GPA patients show increased sensitivity of the B cell receptor signaling pathway compared to HCs [13], possibly making GPA B cells more prone to become activated.

Although both MMF and AZA are used to maintain disease remission, the only controlled study in ANCA-associated vasculitis comparing these drugs showed MMF to be less effective in maintaining disease remission compared to AZA [14]. It has been shown that although MMF was inferior to cyclophosphamide as induction therapy [15,16], MMF-treated GPA patients had a higher relapse rate [16]. This contrasts with treatment outcome in SLE patients in whom MMF seems to be as or even more effective in remission maintenance compared to AZA [17]. In SLE, MMF treatment resulted in a decreased proportion of antigen-experienced B cells (i.e. memory B cells, plasmablasts and plasma cells), reduced serum IgG [18] and diminished *in vitro* B cell IgG and IgM production[19,20]. *In vitro* addition of MPA to a B cell-culture resulted in decreased B- [18,19] and plasma cell formation [18]. Moreover, *in vitro* B cell IL-10 production was dose-dependently inhibited by MPA [19]. Together these findings point to a predominant effect of MMF on the Ab-producing function of B cells in SLE patients. The humoral function of B cells is well known to be an important driver of the SLE pathogenesis [21], whereas this is less clear in GPA. Importantly, little is known about the *in vitro* effects of MPA and 6-MP and the influence of MMF and AZA therapy on B cells of GPA patients.

We hypothesized that the difference in relapse rates between MMF- and AZA-treated GPA patients may be due to drug-related differential effects on B cell subset distribution and/or function. In the current study we assessed the *in vivo* and *in vitro* effects of MMF and AZA on B cells of GPA patients.

## Materials and methods

### Study population

In total, 75 proteinase 3 (PR3)-ANCA positive GPA patients in stable disease remission with or without maintenance immunosuppressive therapy for at least three months were enrolled in this study. The diagnosis of GPA was based on criteria determined in the Chapel Hill Consensus Conference and classification criteria of the American College of Rheumatology were met [22,23]. Remission was defined as absence of clinical disease activity as reflected by a Birmingham vasculitis activity score (BVAS) score of zero. All patients had received induction therapy with cyclophosphamide and corticosteroids, but all treated patients ceased cyclophosphamide treatment at least three months before sampling and switch to treatment with MMF or AZA.

To determine changes in B cell phenotype between treatment groups and *in vitro* B cell cytokine production, MMF- (n = 12) and AZA-treated (n = 13) GPA patients, and age-matched healthy controls (HCs; n = 22)) were included (Treated Cohort 1; Table 1). To assess the *in vitro* effect of the active compounds of MMF (MPA) and AZA (6-MP) on these parameters, we included a second cohort of GPA patients in remission (n = 19), receiving no (n = 18) or only a low dose prednisolone (n = 1) and age- and sex-matched HCs (n = 19; Untreated Cohort 2; Table 1). To determine the *in vitro* effect of MPA and 6-MP on B cell proliferation, we included a third cohort of GPA patients in remission receiving no immunosuppressive medication (n = 10) and matched HCs (n = 11; Untreated Cohort 3; Table 1). To determine *in vitro* B cell proliferation in treated patients, we included a fourth cohort of MMF-treated GPA patients (n = 5), AZA-treated GPA patients (n = 14), both in remission, and the same matched HCs as included in Untreated Cohort 3 (Treated Cohort 4; Table 1).

**Table 1:** Characteristics of HCs and GPA patients for each cohort.

	Treated Cohort 1		Untreated Cohort 2		Untreated Cohort 3		Treated Cohort 4
	HCs	GPA	HCs	GPA	HCs	GPA	GPA
**Subjects, n (% male)**	22 (45.5)	24 (29.2)	19 (47.4)	19 (42.1)	11 (54.5)	10 (50)	19 (42.1)
**Age, mean (range)**	59.5 (39–74)	59.6 (33–78)	54.8 (44–71)	54.6 (28–78)	58.5 (25–83)	55.6 (30–71)	55.4 (31–78)
**cANCA titer, median (range)**		1:40 (0–1:640)		1:40 (0–1:640)		0 (0–1:320)	1:20 (0–1:320)
**Creatinine μmol/L, median (range)**		81 (65–152)		88 (59–190)		82 (53–155)	89 (52–132)
**CRP mg/L, median (range)**		4.2 (0.3–40)		2.9 (0.6–19)		2.8 (0.6–8)	2.1 (0–14)
**IS therapy at time of sampling, n (%)**		25 (100)		1 (5.3)		0 (0)	19 (100)
**AZA, n (%)**		4 (30.8)					5 (26.3)
**AZA + Pred, n (%)**		9 (69.2)					10 (47.4)
**MMF + Pred, n (%)**		11 (100)					5 (100)
**Pred, n (%)**				1 (5.3)			
**Dosage AZA mg/day, median (range)**		76.9 (25–150)					125 (21.5–200
**Dosage MMF mg/day, median (range)**		1615.4 (500–3000)					2000 (1000–2000)

This study was approved by the medical ethics committee of the University Medical Center Groningen (METc no. 2012/ 151), informed written consent was obtained from all patients and the study complies with the Declaration of Helsinki.

### Flow cytometry analysis of B cell subsets

**Fig 1:**
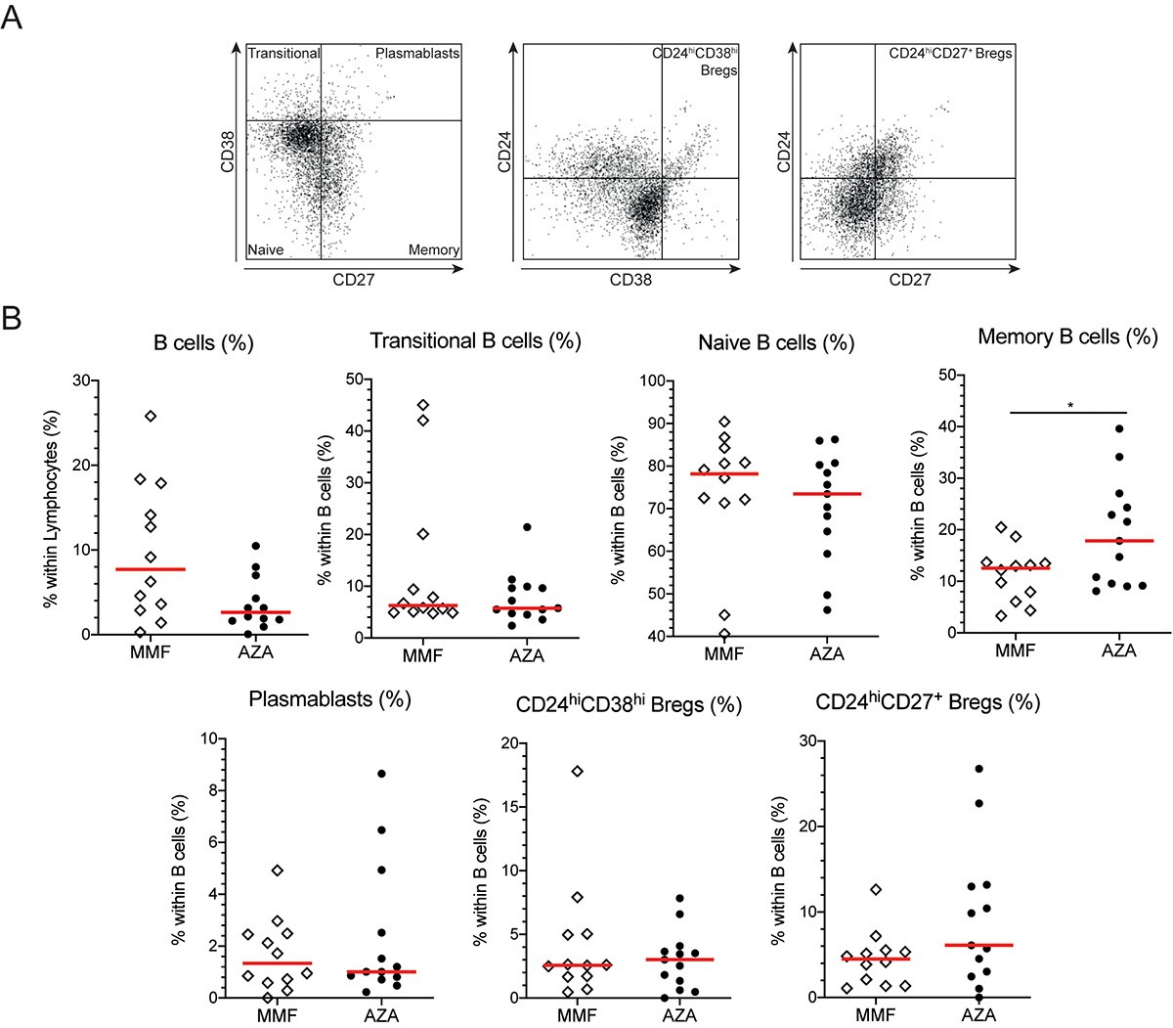
Memory B cells are increased in AZA-treated GPA patients compared with MMF-treated patients. **A.** Representative gating example of the B cell subsets. All gates were set on respective isotype controls. Within CD19^+^ B cells, we first gated on CD38^hi^CD27^-^ transitional B cells, CD38^-/dim^CD27^-^ naïve B cells, CD38^-/dim^CD27^+^ memory B cells and CD38^hi^CD27^+^ plasmablasts using the CD38/CD27 plot. In the CD24/CD38 plot we determined the CD24^hi^CD38^hi^ Bregs and in the CD24/CD27 plots we gated on CD24^hi^CD27^+^ Bregs. **B.** The frequencies of B cells and B cell subsets in MMF- (open diamonds) and AZA-treated (circles) GPA patients. Red lines represent the median value. *p<0.05.

EDTA venous blood was obtained from patients and HCs and immediately washed twice in PBS with 1% BSA (wash buffer). Next, 100 μl blood was incubated with anti-human CD19-eFluor450, CD27-APC-eFluor780, CD38-PE-Cy7 (eBioscience, San Diego, CA, USA), CD24-FITC, IgM-APC, IgD-PE (BD Biosciences, San Jose, CA, USA) or corresponding isotype controls for 15 minutes and treated with 10x FACS Lysing solution (BD Biosciences) for 10 minutes. After washing, samples were acquired on a FACS LSR-II flow cytometer (BD Biosciences). At least 200,000 events were measured and plotted using Kaluza v1.5a flow analysis software (Beckman Coulter, Brea, CA, USA) or FlowJo v10 analysis software (Treestar, Ashland, OR, USA). S1A Fig and Fig 1A show representative gating examples.

### B cell proliferation

**Fig 2:**
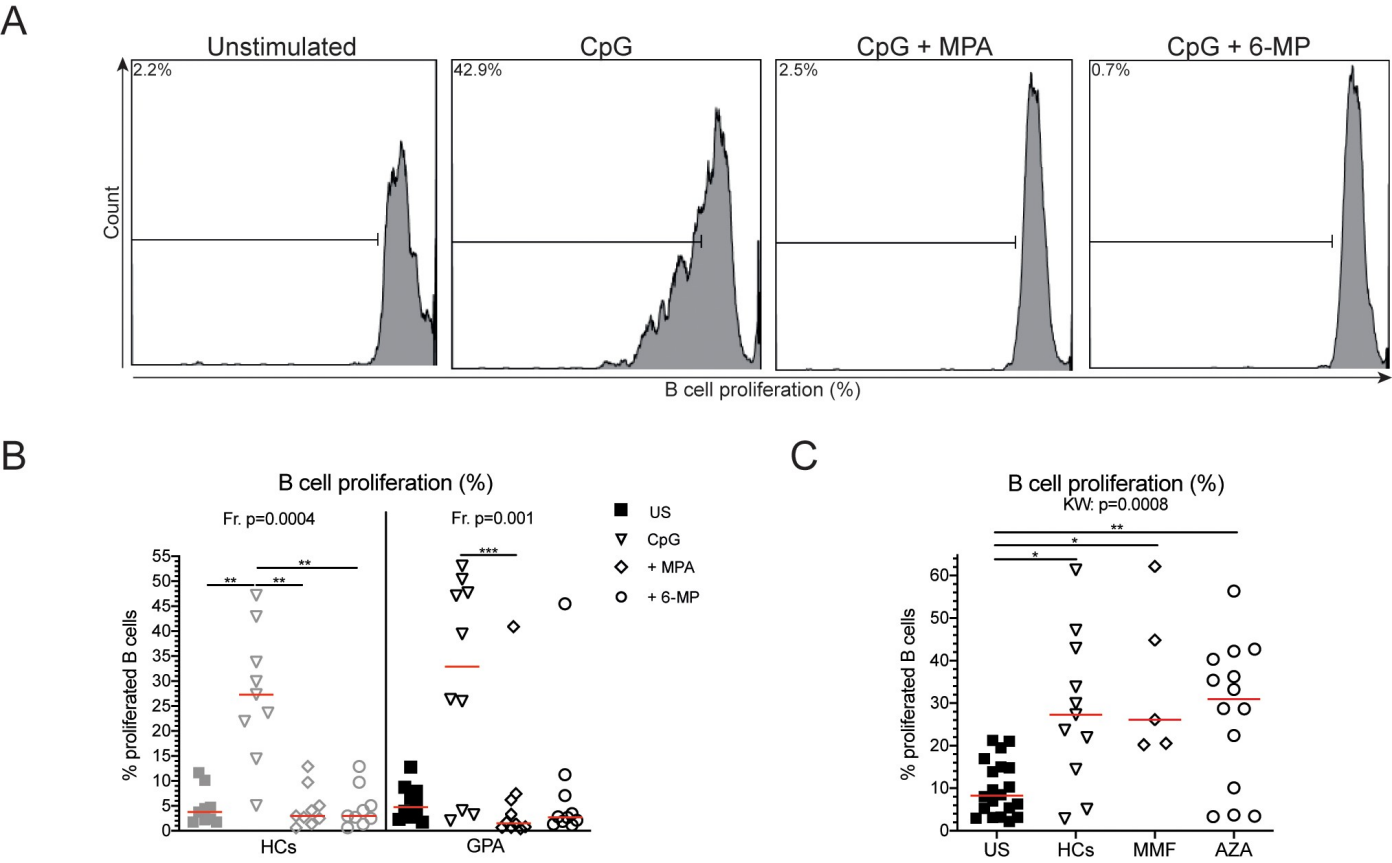
Decreased B cell proliferation upon MPA and 6-MP stimulation. **A.** A representative gating example of proliferating B cells. Within CD19^+^CD22^+^ B cells, B cell proliferation was determined using histograms. Gates were set on the unstimulated sample. **B.** B cell proliferation is given for both HCs and GPA patients for the unstimulated (US; squares), and CpG- (open triangles), MPA+CpG-(open diamonds), and 6-MP+CpG (open circles) stimulated samples. **C.** B cell proliferation is depicted for unstimulated (US) PBMCs (squares), HCs (open triangle), MMF-treated (open diamonds) and AZA-treated patients (open circles). Red lines represent the median value. *p<0.5, **p<0.01, ***p<0.001.

Peripheral blood mononuclear cells (PBMCs) were isolated, frozen and thawed as described before [24]. PBMCs were washed twice in PBS, followed by a 15-minute incubation at 37°C with eFl670 (eBioscience). Next, PBMCs were cultured in the presence or absence of 500 ng/mL CpG (Hycult Biotech, Uden, the Netherlands), with 3 μM MPA or 3 μM 6-MP (Sigma-Aldrich, St. Louis, MO, USA). After 3 days of culture, samples were washed and stained with anti-human CD19-eFluor 450, CD22-APC (BioLegend, San Diego, CA, USA) and CD3-BV786 (BD Biosciences). At least 200.000 cells were acquired and plotted using Kaluza v1.5a or FlowJo v10. In S2 Fig and Fig 2A representative gating examples are given.

### Determination of in vitro B cell cytokine production

To determine the *in vitro* effects of 6-MP and MPA on B cell cytokine production PBMCs were cultured at a concentration of 1*10^6^ cells/mL in the presence of 500 ng/mL CpG alone or in combination with 3 μM MPA or 3 μM 6-MP. After 3 days of culture, PBMCs were restimulated for 5 hours with 2 mM calcium ionophore (Ca-I) and 50 ng/mL phorbol myristate acetate (PMA) in the presence of 10 μg/mL brefeldin A (BFA; Sigma-Aldrich). Next, cells were stained with Zombie Dye NIR (BioLegend) to exclude dead cells. Cells were washed in PBS with 1% BSA (wash buffer) and stained for 15 minutes with anti-human CD19-eFluor 450, CD22-PE-Cy5 (BioLegend), and CD3-BV786. Afterwards, cells were treated with the Fix&Perm kit (Invitrogen, Life Technologies, Grand Island, NY, USA) and incubated with anti-human IL-10-PE, and IL-6-APC and tumor necrosis factor (TNF)-α-AF488 (BioLegend) to stain intracellular cytokines. Samples were acquired on a FACS LSRII and analyzed in Kaluza v1.5a. Gates were set per donor on unstimulated sample. A representative gating example is given in Fig 2A.

**Fig 3:**
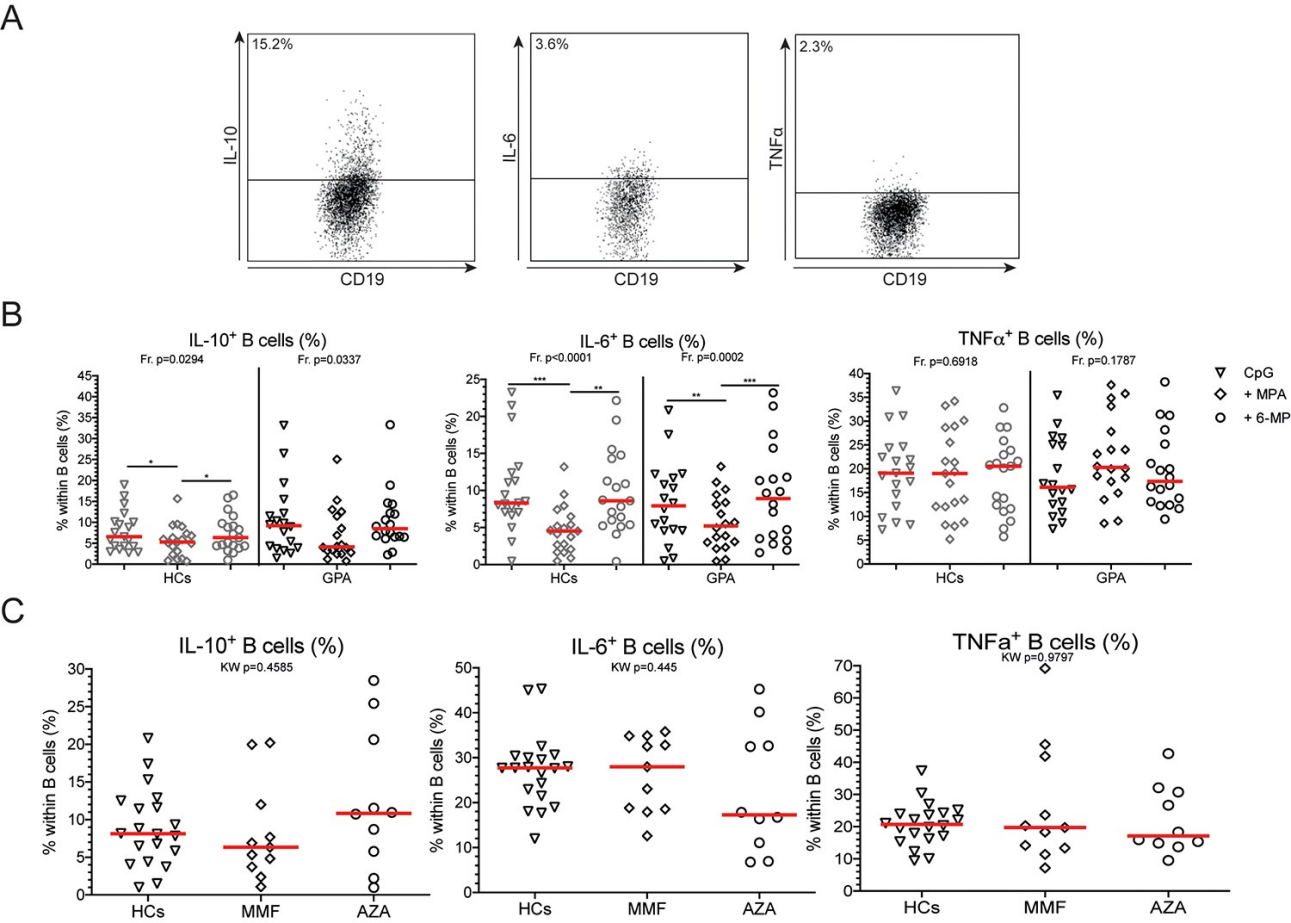
MPA decreases B cell IL-6 production. **A.** Representative gating example of B cell cytokine production. All gates were set on the unstimulated sample for each donor. Within CD19^+^CD22^+^ B cells, IL-10^+^, IL-6^+^ and TNFα^+^ B cells were determined. **B.** The frequencies of all cytokine-positive B cells are depicted for HCs (grey) and GPA patients (black) in CpG only (open triangles), CpG+MPA (open diamonds), and CpG+6-MP (open circles) stimulated PBMCs. **C.** IL-10^+^, IL-6^+^ and TNF⍺^+^ B cell frequencies in HCs (open triangles), MMF-treated (open diamonds) and AZA-treated (open circles) GPA patients. Red lines represent the median value. *p<0.05, **p<0.01, ***p<0.001.

To assess the influence of AZA and MMF on *in vitro* B cell cytokine production, PBMCs were cultured at a concentration of 1*10^6^ cells/mL in the presence of 500 ng/mL CpG or left unstimulated. The same procedures and materials were used for restimulation and staining as described above, except for two monoclonal antibodies: CD22-APC and IL-6-PE-Cy7 (eBioscience). Gates were set per donor on unstimulated sample. Representative gating examples are given in S2 Fig and Fig 3A.

### Statistical analysis

Statistical analysis was performed using Graphpad Prism v7 (GraphPad Software, San Diego, CA, USA). Data are presented as scatter dot plots with median values. Data within the text are represented as median values and range. Normality was tested using the Shapiro-Wilk test. If groups were normally distributed, an unpaired T-test was performed to compare two groups. If all groups were not normally distributed, the Mann-Whitney test was used to compare two groups. The Kruskal-Wallis (KW) test was used to test for differences between three or more groups for unpaired samples, and the Dunn’s multiple comparisons test as a post-test to compare individual groups. A Friedman test was used for paired samples, and the Dunn’s multiple comparisons post-test was used to compare individual groups. P-values of <0.05 were considered statistically significant.

## Results

### No difference in relapse-free survival between mycophenolate mofetil and azathioprine-treated patients

We first compared relapse-free survival between untreated, MMF- and AZA-treated GPA patients. Although both treated patient groups tended to have a reduced relapse-free survival, there was no difference between MMF- and AZA-treated patients (S1B Fig).

### Azathioprine treated GPA patients show an increased frequency of circulating memory B cells

We then determined the frequencies of circulating B cell subsets in HCs and GPA patients that were actively treated with MMF or AZA, and patients not receiving maintenance therapy (untreated and treated patient cohorts 1; Fig 1A). The total B cell frequency was not significantly different between MMF- and AZA-treated patients (Fig 1B). Only the memory B cell population was significantly different between both treated patient groups: AZA-treated patients presented with an increased memory B cell frequency (p = 0.0271; Fig 1B).

In line with previously published research [11], untreated GPA patients showed an increased frequency of naïve B cells (p = 0.0016), whereas the memory B cell frequency was decreased compared with HCs (p = 0.0001; S2A Fig). Untreated patients tended to have an increased frequency of transitional B cells (p = 0.0769), whereas their CD24^hi^CD38^hi^ Breg frequency tended to be decreased compared with HCs (p = 0.0886; S2A Fig).

We also compared the absolute numbers of circulating lymphocytes and B cells between the treated patient groups. No lymphocyte counts were available for the HCs. In the MMF-treated patients the lymphocyte counts were reduced compared with untreated GPA patients (p = 0.0061; S2B Fig). The B cell counts did not different between untreated patients and MMF- and AZA-treated patients (S2B Fig).

### Mycophenolic acid and 6-mercaptopurine inhibit B cell proliferation *in vitro*

To assess the *in vitro* effect of MPA and 6-MP on B cell proliferation, we cultured CpG-stimulated PBMCs of untreated GPA patients (Untreated cohort 3) and HCs in the presence or absence of these compounds (Fig 2A). Both MPA and 6-MP inhibited B cell proliferation in CpG-stimulated PBMCs from HCs compared to PBMCs stimulated with CpG only (Fig 2B). In GPA patients MPA inhibited B cell proliferation in GPA patients, whereas 6-MP tended to do so (Fig 2B).

Subsequently, we determined whether in patients treated with MMF or AZA the capacity of B cells to proliferate differed (Treated cohort 4). As shown in Fig 2C, CpG stimulation of PBMCs from MMF- and AZA-treated patients induced *in vitro* B cell proliferation compared to the unstimulated samples. However, the frequencies of proliferating B cells of MMF- and AZA-treated patients were similar to those observed in CpG-stimulated PBMCs from HCs.

### Mycophenolic acid decreases the frequency of IL-6^+^ B cells *in vitro*

Finally, we determined the effect of MPA and 6-MP on B cell cytokine production *in vitro* in untreated cohort 2. In PBMC cultures of GPA patients and HCs, MPA significantly decreased IL-6^+^ B cell frequencies compared to samples treated with 6-MP, but did not affect the frequencies of TNFα^+^ B cells compared to (Fig 3B). Interestingly, MPA reduced the proportion of IL-10^+^ B cells compared to CpG stimulation in PBMC cultures of HCs but not of GPA patients (Fig 3B). Addition of 6-MP to the PBMC cultures did not affect the IL-10^+^, IL-6^+^ and TNFα^+^ B cell frequencies in GPA patients.

We next assessed the capacity of B cells from MMF- and AZA-treated GPA patients to produce cytokines (Treated cohort 3). As shown in Fig 3C, CpG-stimulated PBMCs from MMF- or AZA-treated patients showed similar IL-10^+^, IL-6^+^ or TNFα^+^ B cell frequencies compared to CpG-stimulated PBMCs from HCs (Fig 3C).

## Discussion

MMF and AZA are drugs that are commonly used to suppress the immune systems of patients with autoimmune diseases. These drugs are effective in maintaining disease remission in GPA patients [14]. Importantly, clinical data indicate that GPA patients treated with MMF are more prone to disease relapses [14,16]. Here, we hypothesized that the previously observed difference in relapse rates between MMF- and AZA-treated GPA patients may be due to drug-related differential effects on B cell subset frequencies and/or functioning. To this end, we assessed whether immunomodulation by MMF and AZA altered B cell subset distribution, and whether the active compounds of these drugs differentially affected B cell functions in GPA patients and HCs.

The increased naïve B cell and decreased memory B cell frequencies in GPA patients compared with HCs is in line with previously published results [11]. Comparing MMF- to AZA-treated patients revealed that the memory B cell frequency was increased in AZA-treated patients, whereas none of the other subsets were different. We subsequently studied B cell proliferation and showed that MPA inhibited *in vitro* B cell proliferation in HCs and GPA patients, while 6-MP only inhibited B cell proliferation in HCs. Moreover, MPA reduced the *in vitro* IL-6^+^ B cell frequency in HCs and GPA patients whereas 6-MP did not. Next, we aimed to confirm these *in vitro* effects on B cell cytokine production by assessing B cell cytokine production and proliferation in PBMC samples from GPA patients receiving MMF or AZA treatment. However, no differential effect on B cell proliferation or cytokine profile was detected between MMF- and AZA-treated patients.

MMF and AZA are known to affect B cell frequencies in the circulation of patients suffering from autoimmune diseases. Eickenberg *et al*. found a decreased transitional B cell frequency in SLE patients treated with AZA compared with MMF-treated SLE patients [18]. We did not find this difference in transitional B cell frequencies in GPA patients. However, in line with our observations, these authors also reported increased circulating memory B cell frequencies in AZA- compared to MMF-treated SLE patients. Eickenberg *et al*. argued that MMF, in contrast to AZA, spared naïve B cells of SLE patients whereas it profoundly decreased antigen-experienced B cells and their functioning (i.e. antibody production and proliferation) [18]. Although we did not assess antibody production, this differential effect of MMF on antigen-experienced B cells might also be responsible for the decreased memory B cell frequencies in MMF-treated GPA patients observed in our study. However, it is unknown whether these differential effects occur due to direct effects of the drugs on B cells or indirectly due to inhibition of innate immune cells or T cells.

An important feature of MMF and AZA is the inhibition of immune cell proliferation. Unexpectedly, we did not observe decreased B cell proliferation in cultured PBMC samples from MMF- and AZA-treated GPA patients (compared with HC) upon CpG stimulation. Previous studies have shown that the active compound of MMF, MPA, is capable of inhibiting T- [4] and B cell proliferation [19] *in vitro* in HCs. MPA was also capable of halting T- and B cell proliferation in patients with active autoimmune hepatitis [25] and SLE [18], respectively. In contrast, 6-MP only inhibited T cell proliferation at high concentrations (>10 nM) [4] or not at all [25]. Here, we show that MPA and 6-MP are both capable of inhibiting B cell proliferation in HCs at a concentration of 3 μM. However, we did not observe differences in the proliferative capacity of B cells from MMF- or AZA- treated patients. It is unclear why these differences were not observed; however, one could speculate that *in vitro* concentrations of the active drug compounds differ from concentrations that are reached in the circulation. In this context, a study in autoimmune hepatitis patients reported blood MPA concentrations of up to 1–3.5 μg/L [25]. In our *in vitro* studies, we used 1 μg/L (= 3 μM) MPA indicating that the applied *in vitro* concentrations are in the range of circulating concentrations reached when patients receive MMF treatment. To date, no data are available for the 6-MP concentrations in the circulation.

We also investigated the effects of both drugs and their active compounds on B cell function by assessing their capacity to secrete cytokines upon CpG stimulation. IL-6 and TNF⍺ production by B cells is considered to promote the inflammatory response. We showed that neither MPA nor 6-MP affected the TNF⍺^+^ B cell frequencies. This is in contrast to others who did show that MPA decreased TNFα production in B cell cultures of HCs [26]. A possible explanation for this discrepancy might be a different set-up of the *in vitro* cultures. Wadia *et al*. cultured B cells in the presence of a stimulation cocktail [26], while we cultured PBMCs and stimulated B cells with CpG. Interestingly, we found that MPA decreased IL-6^+^ B cell frequencies in both GPA patients and HCs, whereas these frequencies were not different in 6-MP treated samples.

The current exploratory study has several limitations. First, only small groups of patients and HCs were included, therefore, our study can be underpowered to detect differences between MMF and AZA. The limited patients included might also explain the lack of difference in relapse-free survival between MMF- and AZA-treated patients in our study. Hence, our findings should be interpreted with caution and preferably be validated in larger patient cohorts. Second, we did not consider differences in induction therapy, which mainly consisted of cyclophosphamide, and its profound effect on the immune system, although included GPA patients were in stable remission for at least 6 months. Third, the *in vitro* concentrations of the active drug compounds, as discussed above, potentially differ from the concentrations that are reached in the circulation. In addition, intracellular cytokine production was measured, and synthesis not necessarily equates secretion. Lastly, we did not assess the effects of both immunosuppressive drugs on serum immunoglobulin or ANCA levels. These could be interesting indicators to link the effect of MMF and AZA on B cell functioning with their differential effect on relapse rate.

In conclusion, we showed that MPA—the active compound of MMF—inhibits, in contrast to 6-MP, *in vitro* pro-inflammatory B cell cytokine production in GPA patients and HCs, while both compounds inhibited B cell proliferation. MMF could be, at least *in vitro*, more effective in inhibiting the pro-inflammatory B cell response of GPA patients compared to AZA. More research is needed to assess the effects of both immunosuppressive drugs on other (pathogenic) immune cells in GPA, to elucidate the immune mechanisms underlying the difference in relapse rates between MMF- and AZA-treated patients and to identify the most effective target for therapy.

## Supporting information

S1 Fig Representative gating strategy for the identification of B cells. **A.** Gates were set on lymphocytes using the FSC-A/SSC-A plot. Within the lymphocytes, CD19^+^ B cells were gates. Within the B cell population, subsets were gated as shown in Fig 1A. **B.** Relapse-free survival in a Kaplan-Meier curve shown for untreated (triangles), and AZA- (circles), and MMF-treated (lines) patients over time (months).
(TIF)

S2 Fig B cell subset distribution in HCs and untreated GPA patients. **A.** The frequencies of B cells and B cell subsets in HCs (pyramids) and untreated GPA patients (circles). **B.** The lymphocyte and B cell counts (x10^6^/L) in untreated GPA patients, MMF- and AZA-treated patients. Red lines represent the median value. **p<0.01, ***p<0.001, ****p<0.0001, #p<0.1
(TIF)

S3 Fig Representative gating strategy used to assess B cell proliferation and cytokine production. Using the FSC-A/SSC-A plot, lymphocytes were gated. Within the lymphocytes, doublets were excluded using the FSC-A/FSC-H plot. Next, live cells were gated using the live/dead/SSC-A plot. Within the live cells, CD3^-^ cells were selected. The CD3- cell population was used to gate on CD19^+^CD22^+^ B cells using the CD19/CD22 plot. Representative gating examples of proliferating and cytokine positive B cells are given in Figs 2A and 3A, respectively.
(TIF)

## List of abbreviations

6-MP: 6-mercaptopurine
Ab: Antibody
ANCA: Anti-neutrophil cytoplasmic autoantibody
AZA: Azathioprine
Breg: Regulatory B cell
BVAS: Birmingham vasculitis activity score
Ca-I: Calcium ionophore
cANCA: cytoplasmic anti-neutrophil cytoplasmic autoantibody
CpG: CpG-oligonucleotides
CRP: C-reactive protein
GPA: Granulomatosis with polyangiitis
HCs: Healthy controls
IL: Interleukin
IMPDH2: Inosine monophosphate dehydrogenase 2
IS: Immunosuppressive
MMF: Mycophenolate mofetil
MPA: Mycophenolic acid
PBMCs: Peripheral blood mononuclear cells
PMA: Phorbol myristate acetate
PR3: Proteinase 3
Pred: Prednisone
SLE: Systemic lupus erythematosus
Th cell: T helper cell
TNF: Tumor necrosis factor
