# Supplementary figures and images for: Mycophenolic acid and 6-mercaptopurine both inhibit B-cell proliferation in granulomatosis with polyangiitis patients, whereas only mycophenolic acid inhibits B-cell IL-6 production
Source: PLoS One. 2020 Jul 9;15(7):e0235743. doi: 10.1371/journal.pone.0235743 (PMC7347169; doi:10.1371/journal.pone.0235743)

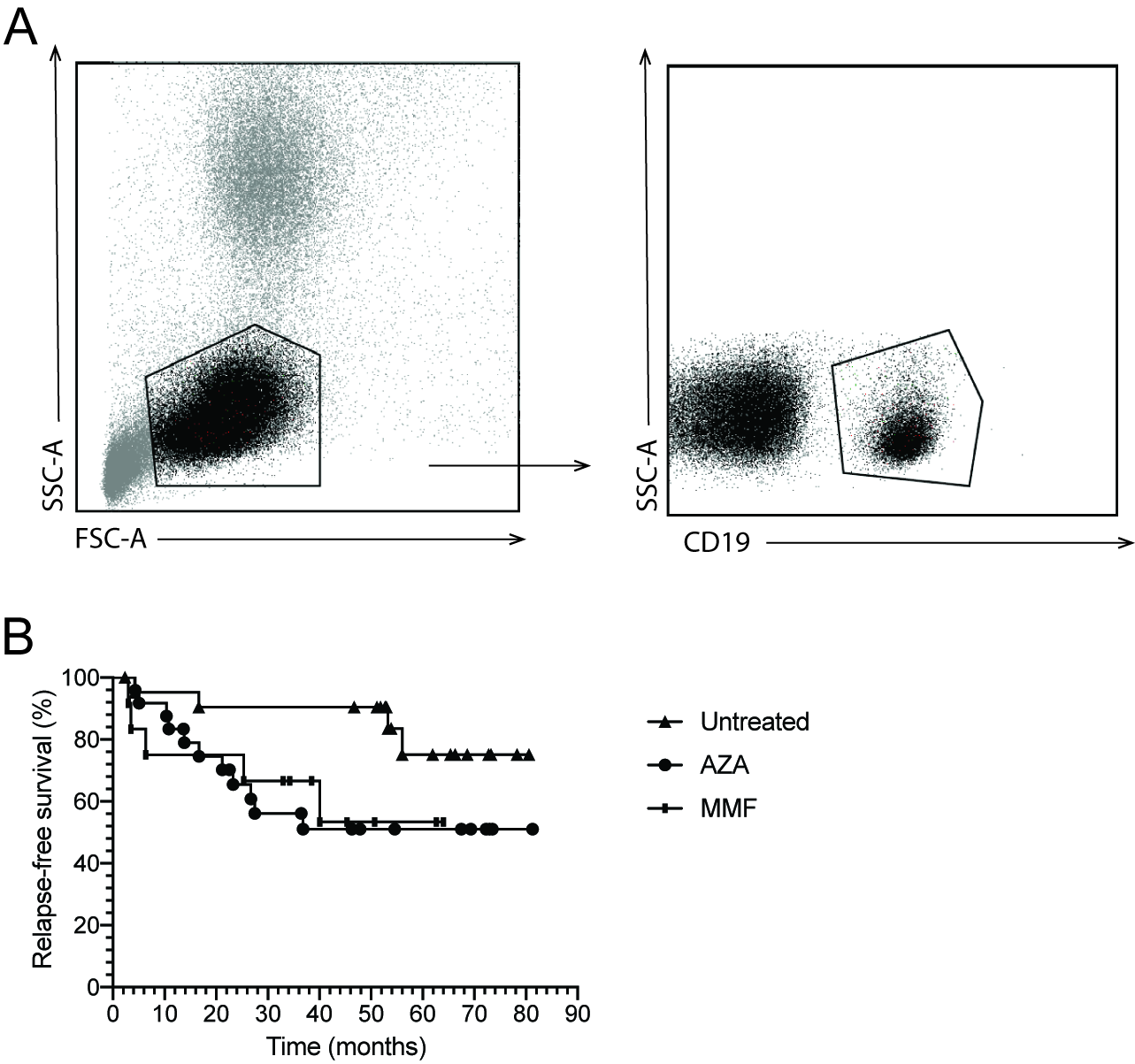

Supplement: S1 Fig — A. Gates were set on lymphocytes using the FSC-A/SSC-A plot. Within the lymphocytes, CD19+ B cells were gates. Within the B cell population, subsets were gated as shown in Fig 1A. B. Relapse-free survival in a Kaplan-Meier curve shown for untreated (triangles), and AZA- (circles), and MMF-treated (lines) patients over time (months). (TIF) [file pone.0235743.s001.tif]

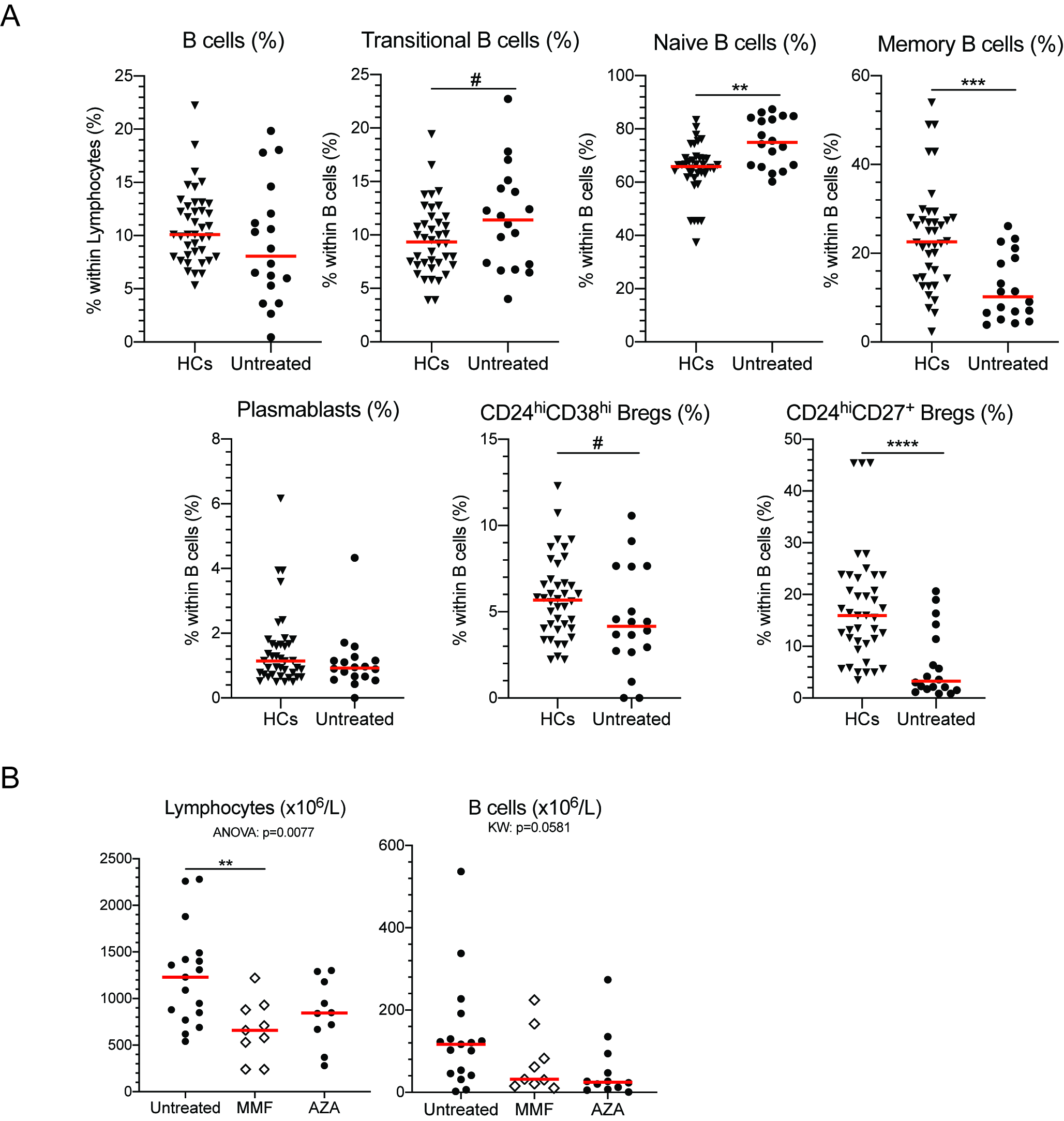

Supplement: S2 Fig — A. The frequencies of B cells and B cell subsets in HCs (pyramids) and untreated GPA patients (circles). B. The lymphocyte and B cell counts (x106/L) in untreated GPA patients, MMF- and AZA-treated patients. Red lines represent the median value. **p<0.01, ***p<0.001, ****p<0.0001, #p<0.1 (TIF) [file pone.0235743.s002.tif]

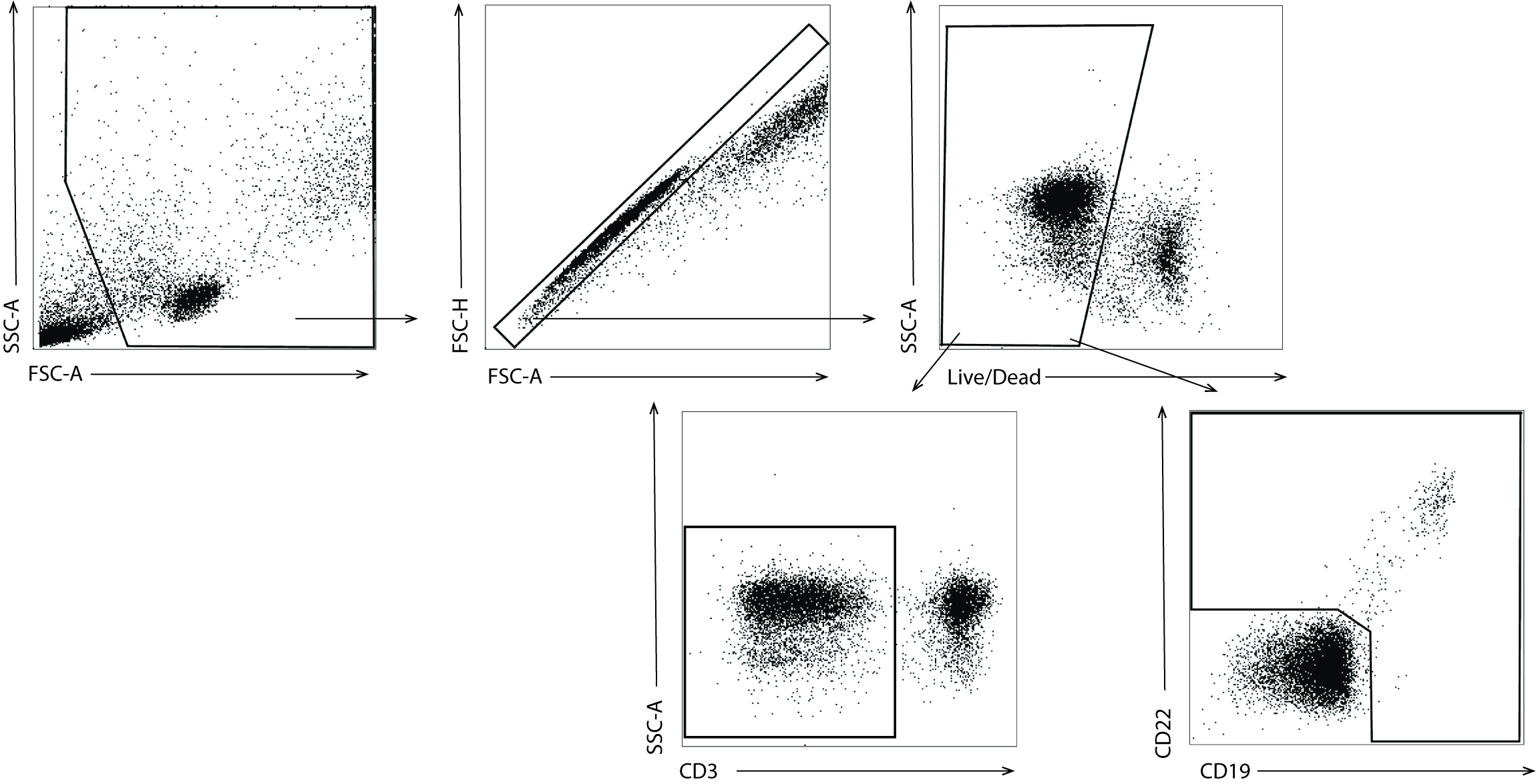

Supplement: S3 Fig — Using the FSC-A/SSC-A plot, lymphocytes were gated. Within the lymphocytes, doublets were excluded using the FSC-A/FSC-H plot. Next, live cells were gated using the live/dead/SSC-A plot. Within the live cells, CD3- cells were selected. The CD3- cell population was used to gate on CD19+CD22+ B cells using the CD19/CD22 plot. Representative gating examples of proliferating and cytokine positive B cells are given in Figs 2A and 3A, respectively. (TIF) [file pone.0235743.s003.tif]
